# Electroanalytical Overview: The Determination of Levodopa (L-DOPA)

**DOI:** 10.1021/acsmeasuresciau.2c00071

**Published:** 2023-02-03

**Authors:** Robert D. Crapnell, Craig E. Banks

**Affiliations:** Faculty of Science and Engineering, Manchester Metropolitan University, Chester Street, Manchester M1 5GD, United Kingdom

**Keywords:** L-DOPA, levodopa, electrochemistry, electroanalytical, sensor, Parkinson’s disease, point-of-care, POC, voltammetry

## Abstract

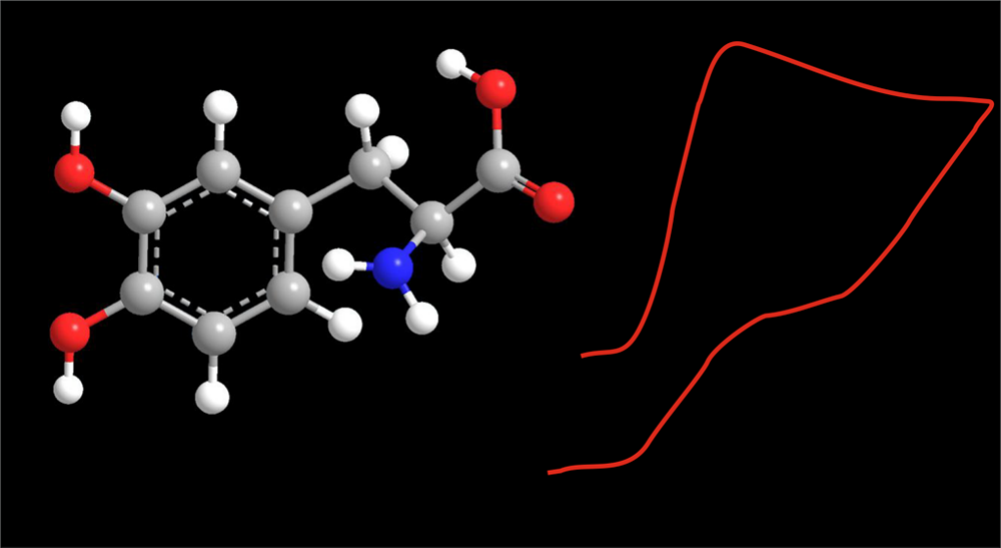

L-DOPA (levodopa) is a therapeutic agent which is the most effective medication for treating Parkinson’s disease, but it needs dose optimization, and therefore its analytical determination is required. Laboratory analytical instruments can be routinely used to measure L-DOPA but are not always available in clinical settings and traditional research laboratories, and they also have slow result delivery times and high costs. The use of electroanalytical sensing overcomes these problems providing a highly sensitivity, low-cost, and readily portable solution. Consequently, we overview the electroanalytical determination of L-DOPA reported throughout the literature summarizing the endeavors toward sensing L-DOPA, and we offer insights into future research opportunities.

## Introduction: L-DOPA

L-DOPA is a naturally occurring isomer of the amino acid 3,4-dihydroxyphenylalanine which was first isolated in 1913 from a seedling of *Vicia faba*; Hornykiewicz provides a thorough historical review on L-DOPA.^1^ L-DOPA is a therapeutic agent, and the precursor to dopamine, and clinicians use L-DOPA as a dopamine replacement agent for the treatment of Parkinson’s disease and dopamine-responsive dystonia.^2^ Parkinson’s disease is reported to be the second most common neurodegenerative disorder after Alzheimer’s disease. It is reported that more than 10 million people globally are living with Parkinson’s disease and more than 1.2 million are within Europe; this is forecast to double by 2030.^3^ L-DOPA is administered with carbidopa for the treatment of Parkinson’s disease where carbidopa prevents the conversion of L-DOPA into dopamine outside the brain, permitting more L-DOPA to reach the brain.^4^ Owing to the short half-life of L-DOPA, ∼90 min, this provides a narrow therapeutic window which can contribute to patients experiencing nonmotor fluctuations and motor symptoms.^5,6^ L-DOPA needs to be sustained at the therapeutic level to avoid low and high doses to circumvent Parkinsonism, anxiety and orthostatic hypotension, dyskinesias, and psychosis.^5^ Overall, there is a clinical need to measure L-DOPA.

Traditionally analytical based laboratory equipment have reported including high-performance liquid chromatography,^7^ gas chromatography/mass spectrometry,^8^ and liquid chromatography–electrospray ionization to name a few.^9^ Although such methods are the most sensitive, akin the “gold standard”, they usually require small sample volume and are not always available in clinical settings, treatment environments, and traditional research laboratories.^7^ Another issue is that such laboratory methods are not a viable methodology for the timely adjustment of L-DOPA dosage due to their slow delivery of the results and high costs.

An alternative approach is the use of electrochemical based sensing strategies which offer rapid, robust, portable electroanalytical solutions.^10^ Electroanalytical approaches exhibit high selectivity and sensitivity when careful attention is given to the electrode materials, sizes, their modification, and the chosen electrochemical technique toward the target analyte.^11^ In this review, we have overviewed the recent advances in the electroanalytical sensing of L-DOPA, and future challenges and opportunities are emphasized.

## Electroanalytical Approaches

Table 1 presents an overview of the electroanalytical detection of L-DOPA; we will consider key highlights below. Blandini et al.^12^ applied the use of high-performance liquid chromatography (HPLC) using electrochemical (coulometric) detection for the quantification of L-DOPA and its metabolite 3-*O*-methyldopa (3-OMD) within plasma. The authors reported that the limit of detection (LOD) for L-DOPA and 3-OMD was 2 and 6 ng/109 platelets, respectively; this was extended to a population of patients with Parkinson’s disease under treatment with L-DOPA.^12^ Rizzo et al.^13^ reported the extension of this approach which they applied to the measurement of the total and the nonprotein-bound fraction of L-DOPA in plasma which exhibited sample runs of less than 5 min. Galal and co-workers^14^ reported sensing of norepinephrine, L-DOPA, epinephrine, and dopamine using HPLC with amperometric detection. The authors reported that the conducting polymer poly(3-methylthiophene) gave detection limits as low as 10^–8^ to 10^–9^ M which were superior to those using platinum or glassy carbon electrodes, 10^–6^ to 10^–8^ M.^14^ This was attributed to the intrinsic catalytic property of the polymer electrode surface toward the redox behavior of the compounds studied.^14^ Dutton et al.^15^ employed ion-pair reverse-phase HPLC with an electrochemical detector for the quantification of L-DOPA within urine and plasma. This approach was applied to therapeutic monitoring of elderly patients with established Parkinson’s disease being treated with L-DOPA. This experimental setup has been applied using HPLC–amperometric detection using a carbon fiber (14 μm diameter) microelectrode flow cell.^16^

**Table 1:** Overview of Various Electrochemical Approaches Reported for the Detection of L-DOPA

electrode	method of detection	linear range	limit of detection	sample medium	comments	ref
gold-SPE	AMP	1–660 μM	0.99 μM	pharmaceutical		(58)
p-Ni^II^TAPc/GCE	AMP	10–0.1 μM	0.1 μM	pharmaceutical		(78)
MWCNT/poly(thionine)/tyrosinase/SPE	AMP	0.8–22 μM	2.5 μM	human serum		(32)
SWCNT-COOH/Nd_2_O_3_–SiO_2_/GCPE	AMP	2–52 μM	0.7 μM	human urine, pharmaceutical		(36)
MWCNT-QD/GCE	AMP	a	a	a		(37)
cobalt hexacyanoferrate/LMCGCE	AMP	0.1–1900 μM	17 nM	human blood serum and pharmaceutical		(79)
nickel hexacyanoferrate/graphite	AMP	0.8–2000 μM	0.53 μM	pharmaceutical		(80)
AuNP/PPy/GCE	AMP	0.1–6.0 μM	0.075 μM	human urine and pharmaceutical		(81)
WO_3_–PEG/GCE	AMP	0.1–1 μM	120 nM	a		(82)
β-cyclodextrin doped poly(2,5-diaminobenzenesulfonic acid)/GC	CV	1–200 μM	0.418 μM	pharmaceutical	in the presence of ascorbic acid	(83)
Ru-red/NaY	CV	120 μM to 10 mM	85 μM	pharmaceutical		(84)
oxovanadium-salen complex^b^/GPE	FIA	1–100 μM	0.8 μM	a		(85)
AME	RDE	0–350 μM	0.23 μM	a		(71)
HRP/MWCNT-pPDA-GCE	DPV	0.1–1.9 μM	40 nM	a		(22)
quercetin/fMWCNT/GCE	DPV	0.90–85.0 μM	0.381 μM	pharmaceutical and yogurt (kefir) samples	in the presence of uric acid and tyramine	(23)
WO_3_NPs/Hb/MWCNT/CPE	DPV	60–1070 μM	0.25 μM	human urine and serum	in the presence of uric acid and folic acid	(24)
graphene/ZnONF/ITO	DPV	1–60 μM	1 μM	human urine		(86)
3D HGB/Ni NP/ITO	DPV	1–60 μM	0.4 μM	human urine		(87)
Fe_2_O_3_NP-MWCNT/GCE	DPV	0.3–8 μM	0.24 μM	pharmaceutical		(25)
ZnO NR-GF/ITO	DPV	5–50 μM	5 μM	human urine		(88)
chloranil/CPE	DPV	3–500 μM	0.65 μM	human urine	in the presence of benserazide	(89)
GF/ITO	DPV	0.05–40 μM	20 nM	human urine		(90)
cysteic acid–GCE	DPV	0.65–22 μM	0.2 μM	human serum	in the presence of l-tyrosine and uric acid	(91)
ferrocenedicarboxylic acid/CNT/CPE	DPV	0.04–1100 μM	12 nM	human urine, well and tap water	in the presence of NADH and tryptophan	(26)
Au–Pd NP/NPSS	DPV	5–55 μM	0.2 μM	human urine and serum, pharmaceutical	in the presence of uric acid	(92)
AuNP/titanium dioxide nanotubes	DPV	10–70 μM	a	pharmaceutical		(93)
graphene nanoribbons/SPE	DPV	10–50 μM	a	human urine	in the presence of ascorbic acid and uric acid	(46)
N-GE/NiO	DPV	0.03–386.8 μM	17 nM	vegetable (sweet potato)		(47)
AuNP–CNT/PGE	DPV	0.1–150 μM	50 nM	pharmaceutical		(27)
PbO_2_/CPE	DPV	260–1200 μM	25 μM	pharmaceutical		(94)
SWCNT/GCE	DPV	0.5–20 μM	0.3 μM	a		(28)
polypyrrole/CNT/GCE	DPV	1–100 μM	0.1 μM	a		(95)
activated screen-printed carbon electrode	DPV	1–100 μM	0.47 μM	pharmaceutical	in the presence of benserazide	(62)
poly(methyl orange)/CPE	DPV	10–800 μM	3.69 μM	pharmaceutical		(96)
graphene/GCE	DPV	0.04–79 μM	22 nM	mouse brain extract and pharmaceutical		(52)
TNF/GO/GCE	DPV	0.3–60 μM	15.9 nM	human cerebrospinal fluid (CSF), blood serum (BS) and plasma (BP)		(97)
cobalt porphyrin/TiO_2_/CPE	DPV	0.1–100 μM	62 nM	drinking water, human urine, human blood serum	in the presence of carbidopa	(66)
2,7-bis(ferrocenyl ethyl)fluoren-9-one/CNT/CPE	DPV	0.1–700 μM	58 nM	well water, human urine	in the presence of uric acid and folic acid	(21)
Co(OH)_2_ NP/MWCNT-CILE	DPV	0.25–225 μM	0.12 μM	human blood serum	in the presence of serotonin	(29)
MWCNT/chitosan/GCE	DPV	2–220 μM	0.12 μM	human blood serum and urine	in the presence of serotonin	(30)
MWCNT/poly(Evans blue)/GCE	DPV	0.5–100 μM	0.53 μM	human blood serum, pharmaceuticals	in the presence of serotonin and folic acid	(31)
reactive blue 19/MWCNT/CE	DPV	1.37–92.59, 92.59–833.33 μM	0.37 μM	human urine, pharmaceutical	in the presence of ascorbic acid, insulin and uric acid	(33)
polyglycine/ZnO NP/MWCNT/CPE	DPV	5–500 μM	0.08 μM	human urine, pharmaceutical	in the presence of ascorbic acid	(34)
PEDOT/SWCNT/GCE	DPV	0.1–20 μM	0.1 μM	a		(35)
AuNP/PGE	DPV	20–100 μM	1.54 μM	*Mucuna pruriens* seeds (MPS) and leaves (MPL) and commercial Siddha product (CSP)	in the presence of ascorbic acid	(98)
poly(xylene cyanol)/CPE	DPV	20–9000 μM	1.8 μM	pharmaceutical		(99)
3,4′-AAZ/ZnO NP/CPE	DPV	0.1–700 μM	0.03 μM	pharmaceutical, human blood serum, and drinking water	in the presence of carbidopa	(67)
graphene/GCE	DPV	1–16 μM	0.8 μM	a	in the presence of carbidopa	(48)
GR/DE/IL/CPE	DPV	0.015–1000 μM	5 nM	pharmaceutical, human urine and blood serum		(100)
CPE	DPV	a	a	pharmaceutical		(19)
polyaniline/SPEs	DPV	0.1–1 mM	10 μM	a		(61)
CuNPs/MWCNT/MIP/GCE	DPV	0.01–1 μM	7.2 nM	pharmaceutical, human urine		(38)
graphene–MIP/GCE	DPV	0.4–100 μM	12 μM	pharmaceutical, human blood serum		(50)
Gd-ZnO nanoflower/GO/GCE	DPV	10–100 nM	0.82 nM	pharmaceutical, human urine		(101)
FeTiO_2_/CPE	DPV	a	a	a		(102)
FeSnO_2_/CPE	DPV	0.7–100 μM	a	a		(103)
PCFCuNP/GE	DPV	0.2 μM to 1.0 mM	0.06 μM	pharmaceutical		(104)
SWCNT–chitosan–IL/GCE	DPV	2–450 μM	a	human serum and urine	in the presence of acetaminophen	(40)
GP-CAc/PVC	SWV	8–100 μM	0.06 μM	pharmaceutical		(105)
Co(DMG)_2_ClPy-MWCNT/BPPG	SWV	3–100 μM	0.86 μM	pharmaceutical		(106)
RGO/MOF/PE	SWV	0.1–85 μM	0.02 μM	human urine; tablet		(45)
vinylferrocene/CNT/GCE	SWV	1.0 × 10^–7^ to 6.0 × 10^–4^ M	50.0 nM	human urine		(39)
dysprosium nanowire/CPE	SWV	10 nM to 1 μM	4 nM	pharmaceutical, human serum and urine		(107)

a Not reported.

b *N*,*N*′-ethylene-bis(salicylideneiminato) oxovanadium).

Upon inspection of Table 1, we can see that various researchers have explored bare (solid) electrodes for the sensing of L-DOPA such as boron-doped diamond,^17^ which was applied in extracts from the seeds of velvet bean (*Mucuna prurita* Hook or *Mucuna pruriens*), carbon discs, which were evaluated in the presence of benserazide and (*R*,*S*)-2-amino-3-hydroxypropanohydrazide,^18^ or carbon paste electrodes.^19^ People have adapted their electroanalytical approach where the majority have explored modified electrodes,^20^ such as modification with carbon nanotubes (multiwalled/single-walled).^21−41^

This approach is due to carbon nanotubes (CNTs) arising as a novel nanomaterial following their discovery by Ijima,^42^ where their major role in electrocatalysis underpinned many different applications in sensors. For example, Yan and co-workers^28^ explored a single-walled CNT modified glassy carbon (GC) electrode for L-DOPA detection, and they reported that the “modified electrode exhibited good promotion of the electrochemical reaction of L-DOPA and greatly increased the standard heterogeneous rate constant”.^28^ The authors demonstrated that the sensing of L-DOPA was possible over the range of 0.5 to 20 μM with a LOD reported to be 0.3 μM. That said, there is no evidence of CNTs being electrocatalytic which is attributed to a decrease in the peak-to-peak separation at the CNT modified electrode over that observed at the bare/supporting electrode. It is noted that such comparisons are valid if both the bare and modified electrodes have similar mass transport regimes since the peak potential reflects a point of balance between the electrode kinetics and the rate of mass transport;^43^ Compton et al. provide a thorough overview of the use of CNTs as “electrocatalysts” which is a must read.^43^

Researchers have changed their approach to the use of graphene (reduced graphene oxide) modified electrodes for the sensing of L-DOPA.^44−50^ Graphene is a 2D nanoscale single-atom-thick, sp^2^-bonded material which was rediscovered in 2004 by Novoselov and Geim^51^ and, of course, has attracted extensive attention from electrochemists. For example, Arvand and Ghodsi^52^ reported the development of a graphene modified GC electrode toward the sensing of L-DOPA which gave rise to a linear range of 0.04 to 79 μM and a LOD of 22 nM. This was successfully applied to sensing within a mouse brain extract and a pharmaceutical. The reason for the use of graphene was that “better performance of graphene/GC electrode may be due to the nanometer dimensions of the graphene, the electronic structure, and the topological defects present on the graphene surfaces”.^52^ Indeed, if correctly fabricated, that is not being pristine but having edge plane defect sites, this is highly likely.^53,54^

**Figure 1:**
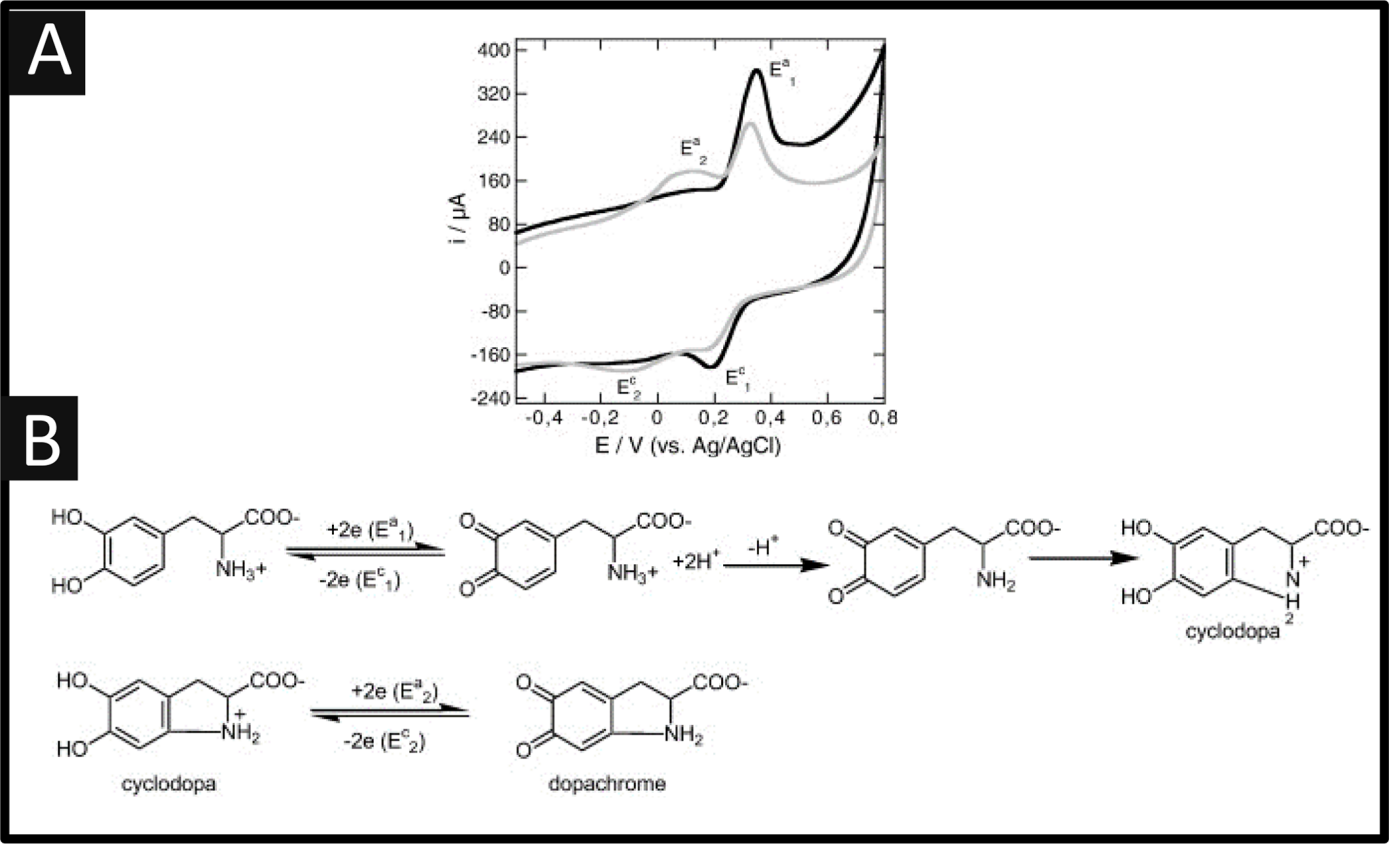
(A) Cyclic voltammograms of an aqueous solution of L-DOPA (1 mM) in KCl (0.1 M, pH = 7.2) recorded on graphene nanosheets deposited on glassy carbon. Scan rate = 50 mV s^–1^; first scan, black line; 20th scan, gray line. (B) Electrochemical mechanism of L-DOPA oxidation. Reprinted with permission from ref (48). Copyright 2013 Elsevier.

The electrochemical sensing of L-DOPA is well established; for example, the electrochemical oxidation of L-DOPA is exemplified within Figure 1A which shows the electrochemical behavior recorded on a graphene nanosheet modified glassy carbon, depicting the first and 20th voltammetric scans which provide insights into the mechanism of L-DOPA.^48^ In the case of the first scan, an electrochemical peak is observed *E*_a1_ = +0.34 V, which is the oxidation of L-DOPA to an open-chained quinone, as shown within Figure 1A. In the case of the 20th scan, a new redox couple becomes evident (*E*_a2_ = +0.09 V, *E*_c2_ = −0.12 V) which is indicative of a slow chemical follow up reaction after the oxidation of L-DOPA; this has been seen with the use of bare glassy carbon electrodes.^55^ Note that at a neutral pH, sufficient unprotonated quinones are available to favor the cyclization reaction while the second redox couple corresponds to the oxidation of the cyclized product, cyclodopa to dopachrome (Figure 1B).^48^

Other work utilizes a composite modified electrode, common throughout the literature in electroanalytical sensing,^56^ comprising many items to help promote the sensing of L-DOPA. For example, Đurđić and co-workers^36^ demonstrated the development of a sensor based on carboxylated single-walled carbon nanotubes (SWCNT-COOH) which have been modified with SiO_2_ coated Nd_2_O_3_ nanoparticles for the sensing of L-DOPA.

Note that SWCNTs have unique physical and chemical properties, such as electrical and thermal conductivity, mechanical strength, and flexibility, and they are ultralight weight; some of these aspects make them useful as the supporting material for metal nanoparticles, but they have suffered in the past due to metallic impurities.^57^ The authors carboxylate the SWCNTs by using a mixture of sulfuric and nitric acids which were subjected to 2 h of ultrasonication and then fluxed for 12 h. Following this, the suspension was cooled and centrifuged and washed with distilled water and then dried for 6 h in a vacuum oven. In terms of SiO_2_ coated Nd_2_O_3_ nanoparticles, the procedure utilized cyclohexane solution containing polyoxyethylene (10) tridecyl ether, which was used to form reverse micelles (solution 1). Another solution containing a suspension of Nd_2_O_3_ nanopowder in ultrapure water (solution 2) alongside tetraethyl orthosilicate was diluted in NH_3(aq)_ (solution 3). Solution 2 was added into a preheated to 50 °C solution 1, while solution 3 was added to this mixture, after which the formed suspension was exposed to hydrolysis (50 °C, 1 h).^36^ Subsequently, the precipitate, comprising SiO_2_ coated Nd_2_O_3_ nanoparticles, was centrifuged, washed three times with propanol, and dried (80 °C, 24 h). Next, the calcination of the dried precipitate was performed within an airflow dryer at 400 °C for 5 h. The final nanocomposite was prepared by placing SWCNT-COOH and Nd_2_O_3_–SiO_2_ nanoparticles in DMF. After 6 h of ultrasonication, DMF was evaporated, and the nanocomposite was dried at 70 °C within a vacuum oven.

**Figure 2:**
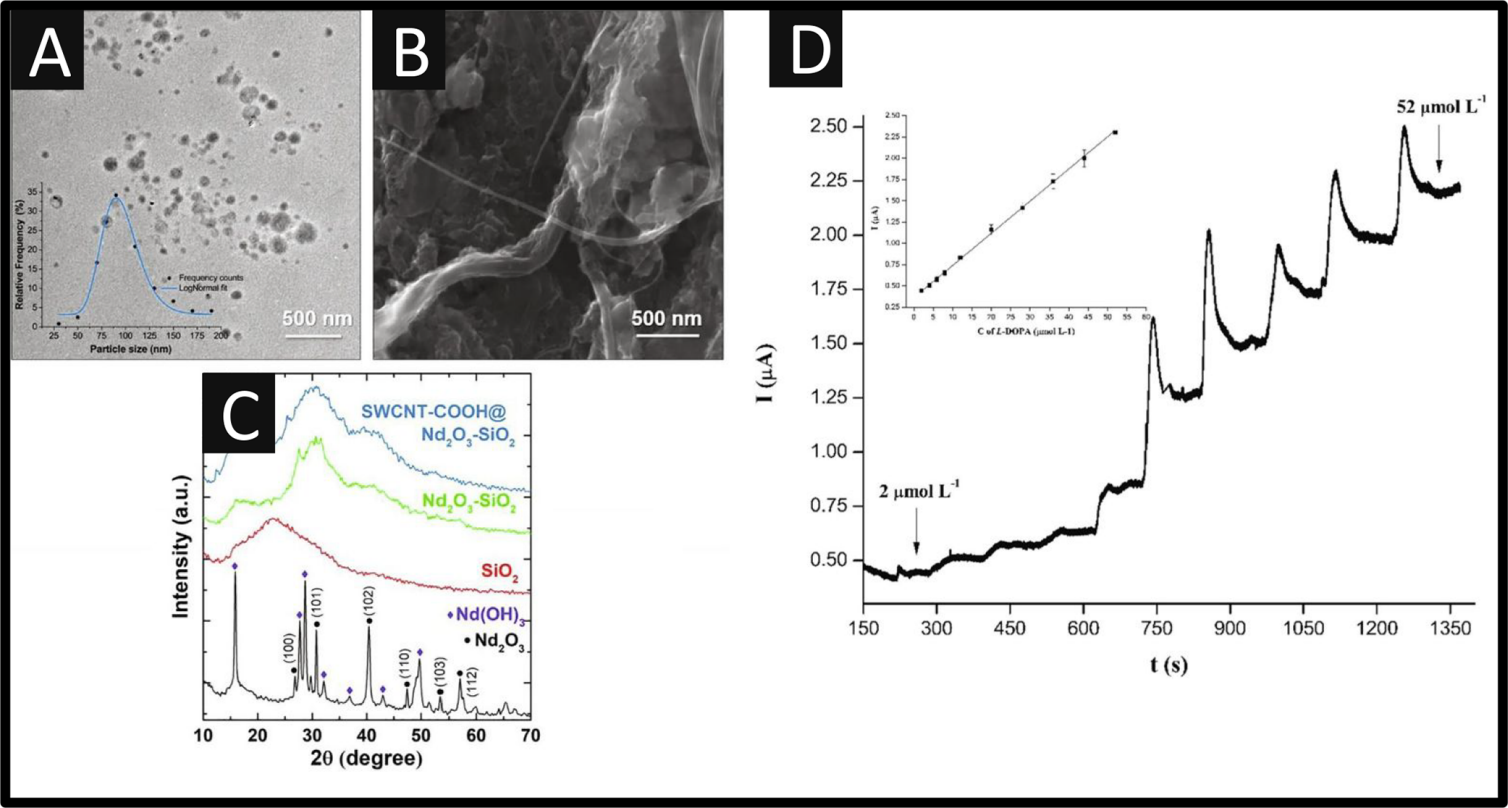
(A) TEM image of Nd_2_O_3_, (B) SEM image of SWCNT-COOH@Nd_2_O_3_–SiO_2_, and (C) respective XRD patterns of the synthesized materials. Inset of panel A, log-normal size distribution of Nd_2_O_3_ nanoparticles. (D) Amperometric *i*–*t* curve for the successive addition of different aliquots of 2 mmol L^–1^ L-DOPA at GCp/SWCNT-COOH@Nd_2_O_3_–SiO_2_ electrode in 0.1 mol L^–1^ PBS (pH = 7.40) at a constant potential of 0.4 V. Inset shows the calibration plot. Reprinted with permission from ref (36). Copyright 2021 Elsevier.

Figure 2A shows a transmission electron microscopy (TEM) image taken of the Nd_2_O_3_, and a scanning electron microscopy (SEM) image of the SWCNT-COOH@Nd_2_O_3_–SiO_2_ is shown in Figure 2B. The former demonstrates that the Nd_2_O_3_ nanoparticles are spherically shaped and well-dispersed with a reported average particle size of *D*_TEM_ = 94 ± 20 nm. Figure 2C displays the X-ray powder diffraction (XRD) spectra for the SWCNT-COOH@Nd_2_O_3_–SiO_2_, which shows that the Nd_2_O_3_ (black line) is a combination of Nd_2_O_3_ and the Nd(OH)_3_ reflections, while the red line reveals a broad peak at 22.5° 2θ which can be allocated to amorphous nanosilica. The other two patterns, green and blue lines, are a combination of the previous, which could be assigned to the amorphous nature of SWCNT-COOH@Nd_2_O_3_–SiO_2_. As shown within Figure 2D, the sensing performance of the SWCNT-COOH@Nd_2_O_3_–SiO_2_ toward L-DOPA exhibited a linear range from 2 to 52 μM with a LOD reported to be 0.7 μM. The sensor was evaluated within a pharmaceutical product, where the results agreed well with the declared dose, and in human urine samples, which exhibited good recoveries over the range of 94–102%.

Naghian and co-workers^45^ reported a metal–organic framework (MOF) that was mixed with reduced graphene oxide to produce an electrochemical sensor that has a high surface area, coupled with improved electrode transfer kinetics. The sensor was able to show a linear range of 0.1 to 85 μM with a LOD of 0.02 μM. The sensors exhibited no interference from such competitive analytes when applied to measuring L-DOPA in spiked human urine and within a pharmaceutical tablet showing recovery rates of 94.0–102.0%, with the RSD value for each sample being less than 3.0%.

**Figure 3:**
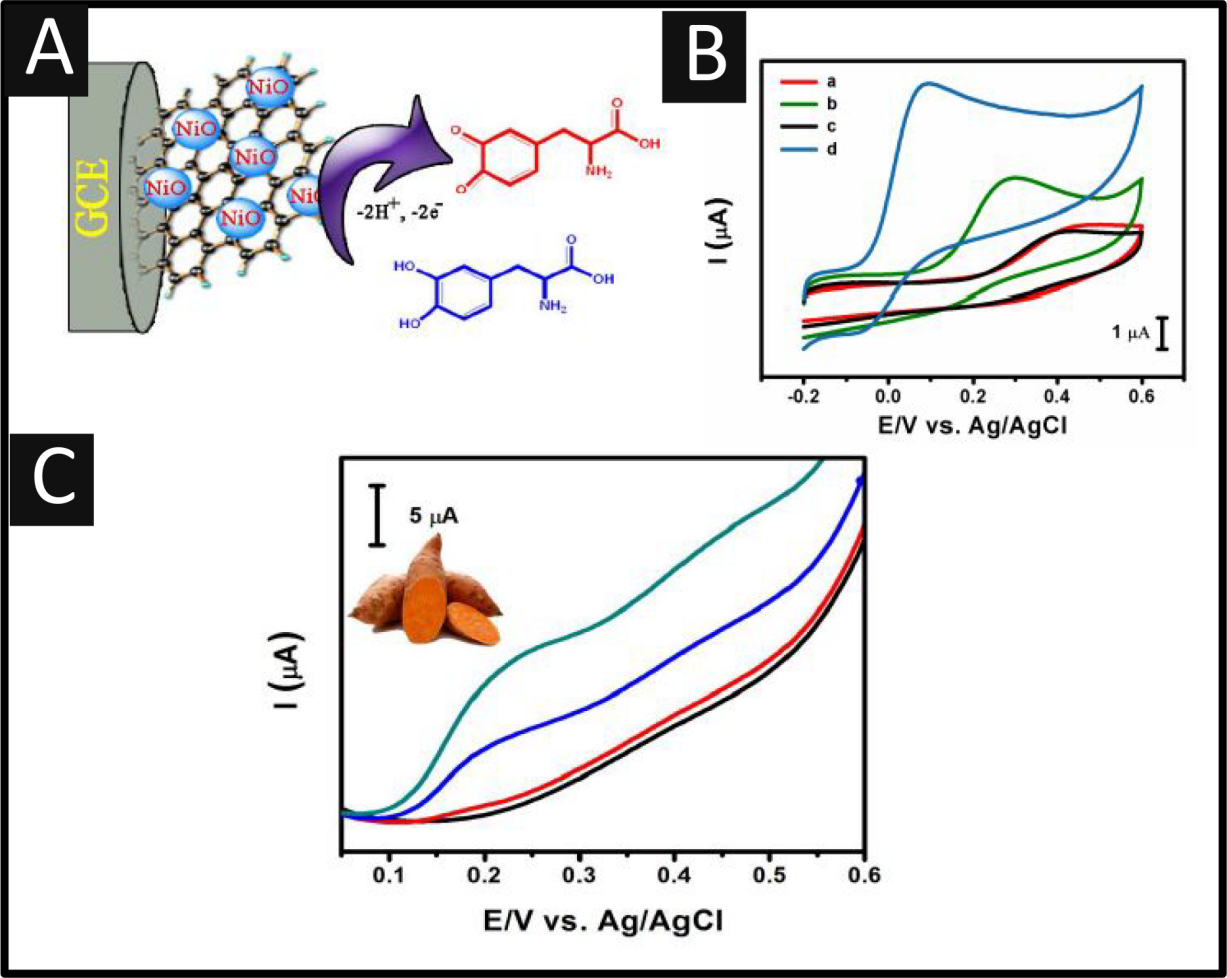
(A) Graphical illustration of nitrogen-doped graphene supported with nickel oxide (N-GE/NiO) nanocomposite supported upon a glassy carbon electrode. (B) Cyclic voltammetric response of bare GCE in the presence of (a) L-DOPA or (b) NiO, (c) GO, and (d) N-GE/NiO/GCE with 200 μM L-DOPA in 0.05 M PBS (pH 7). Scan rate: 50 mV s^–1^. (C) DPVs of the N-GE/NiO/GCE in sweet potato juice sample spiked by L-DOPA with PBS (pH 7). Scan rate: 50 mV s^–1^. Reprinted with permission under a Creative Commons License from ref (47). Copyright 2018 ESG.

Renganathan and co-workers^47^ reported the novel fabrication of nitrogen-doped graphene supported with nickel oxide (N-GE/NiO) nanocomposite which was immobilized upon a GC electrode (Figure 3A). This approach required the fabrication of graphene oxide prepared by the modified Hummers method, where nickel oxide was mixed and kept under continuous stirring for 1 h at a room temperature, after which urea was added as the reducing agent.^47^ This mixture was placed into a Teflon-lined autoclave with a stainless-steel shell (180 °C for 2 h), after which, the precipitate was washed and then dried overnight. Last, the final composite was achieved by calcination in a flowing argon atmosphere by heating at a rate of 10 °C min^–1^ to 400 °C at 2 h. The composite was immobilized upon a glassy carbon electrode. As shown in Figure 3B, the N-GE/NiO exhibited an optimized voltammetric response which was attributed by the authors to the higher electron transfer capacity compared to a bare GC electrode, NiO, and graphene oxide.^47^ The electroanalytical response demonstrated a linear range of 0.03 to 386.8 μM with a LOD of 17 nM. The authors were able to show a novel application and measured L-DOPA in sweet potato juice, shown in Figure 3C, where their N-GE/NiO nanocomposite was suited to measuring L-DOPA with recoveries of 97.8–101.1%.

Another notable approach is that reported by Bergamini and co-workers, who have described the first use of gold screen-printed electrodes (SPEs) for the measurement of L-DOPA.^58^ SPEs are highly useful because differing from classic electrode platforms they can be used as single-shot, disposable, reproducible, and ready to use electrodes. On the other hand, classic (solid) electrodes such as glassy carbon, edge plane and basal plane pyrolytic graphite, or highly ordered pyrolytic graphite need to be rigorously polished and cleaned before undertaking every measurement and require the presence of external reference electrode (RE) and counter electrode (CE). SPEs offer a disposable, reproducible, and low-cost electrode which is easily modified for electrochemical sensor platforms.^59,60^ Though SPEs have inherent advantages, there are limited reports of their use toward the sensing of L-DOPA.^32,46,58,59,61,62^ The authors of ref (58) demonstrated that the sensor was able to measure L-DOPA within 1 to 660 μM with the LOD reported to be 0.99 μM. The method was successfully applied to the determination of L-DOPA in two commercial dosage forms without any pretreatment, which resulted in 104% and 108% recoveries of L-DOPA. Work around this theme reported the first disposable electrochemical biosensor for L-DOPA determination in undiluted serum samples. This approach utilized an outer cross-linked layer containing tyrosinase on the top of a carbon nanotube (CNT) modified SPE/polythionine film. This sensor was able to measure L-DOPA over the range of 0.8 to 22 μM with the LOD reported to be 2.5 μM; the use of a reagent layer enhances sensitivity and stability, decreasing the detection limit of L-DOPA in undiluted serum samples.^32^

**Figure 4:**
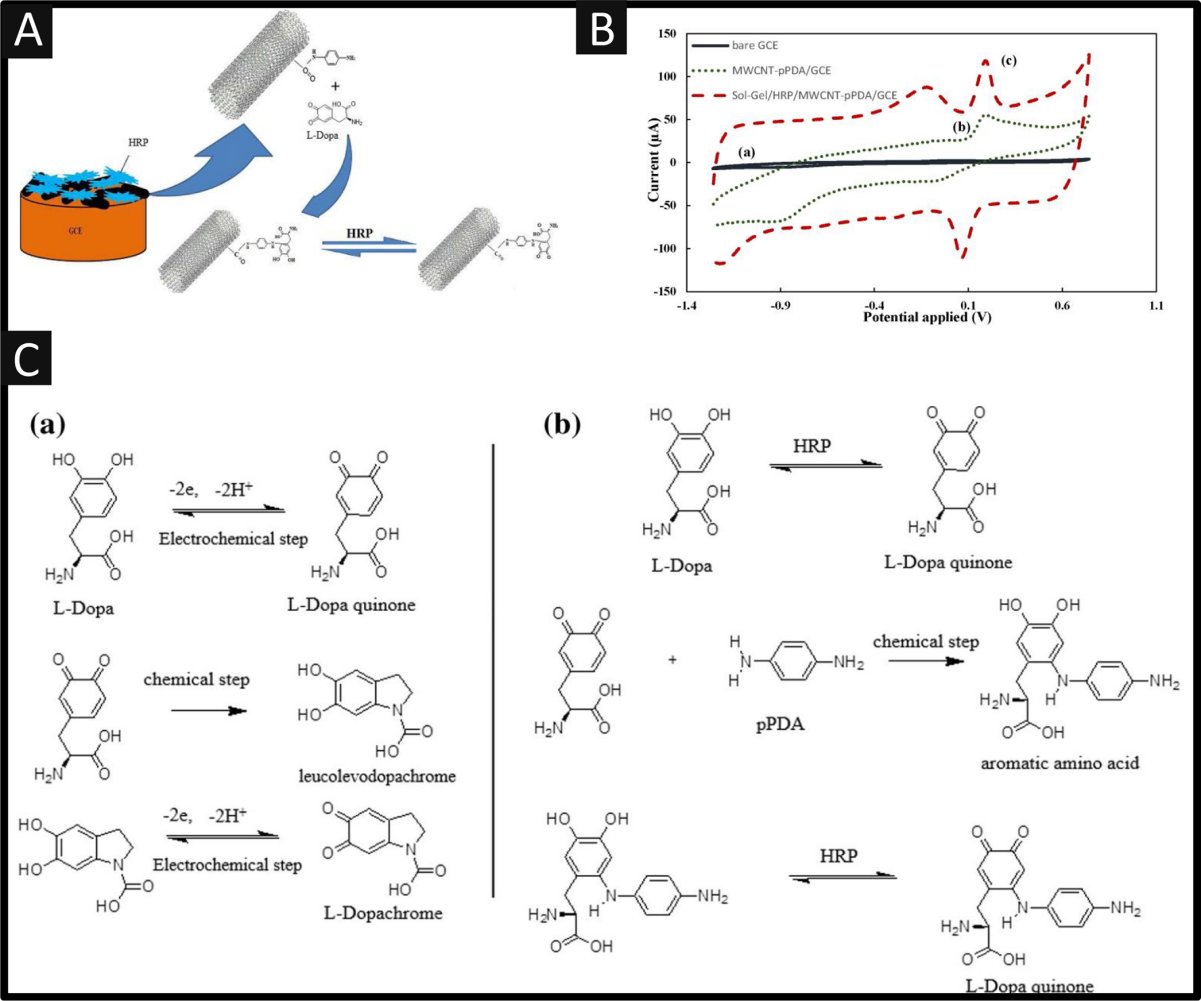
(A) An overview of the sensing of L-DOPA utilizing horseradish peroxidase (HRP), multiwalled carbon nanotubes (MWCNTs) and p-phenylenediamine (pPDA) immobilized upon glassy carbon; (B) cyclic voltammograms of (a) bare GCE, (b) MWCNT-pPDA/GCE, and (c) sol gel/HRP/MWCNTpPDA/GCE in PBS solution (50 mM, pH = 7) containing 50 mM H_2_O_2_ and 2 μM L-DOPA at scan rate of 100 mV/s; (C) possible mechanism for electro-oxidation of L-DOPA in the (a) absence and (b) presence of HRP and pPDA as nucleophile on a modified GCE in PBS solution (50 mM, pH = 7). Reprinted with permission from ref (22). Copyright 2015 Elsevier.

Shoja and co-workers^22^ have reported a biosensor for the measurement of L-DOPA using a horseradish peroxidase/organic nucleophilic-functionalized carbon nanotube composite; see Figure 4A. This biosensor was made via physically immobilizing horseradish peroxidase (HRP) as a catalyst through a sol–gel approach upon the surface of a GC electrode, which was already modified with p-phenylenediamine (pPDA) as an organic nucleophile chemically bonded with functionalized (carboxylic groups) multiwalled carbon nanotubes (MWCNTs). As shown in Figure 4B, typical cyclic voltammograms are recorded in the presence of L-DOPA where there is no redox peak at the bare GC electrode but a smaller, broader redox peak around +0.19 V for anodic peak and −0.09 V for cathodic peak was observed using the MWCNT-pPDA/GCE. Also shown is the response of sol–gel/HRP/MWCNT-pPDA/GCE, which exhibits an anodic peak at +0.19 V and a cathodic peak at +0.08 V. The authors^22^ reported that the high electrocatalytic activity for oxidation of L-DOPA is related to the simultaneous presence of HRP as electrocatalyst mediator and MWCNT-pPDA as a nucleophilic composite, which is coupled with a high surface area and good conductivity from the presence of MWCNTs. Figure 4C shows the electrochemical mechanism of the oxidation of L-DOPA, but in the presence of the pPDA, the scheme shows that in the first step, L-DOPA is oxidized to L-DOPA quinone by HRP which then undergoes an 1,4-Michael addition with pPDA as a nucleophile leading to a respective aromatic amino acid in the second step. This aromatic amino acid, which is formed in the second step, is an electroactive intermediate and is subsequently oxidized to a respective quinone by HRP in the third step.^22^ The authors were able to show that their composite achieved the determination of L-DOPA from 0.1 μM to 1.9 μM, with a low LOD of 40 nM, but only in model solutions; despite this the authors noted that their sensor has advantages such as rapid response, high stability, and reproducibility.

**Figure 5:**
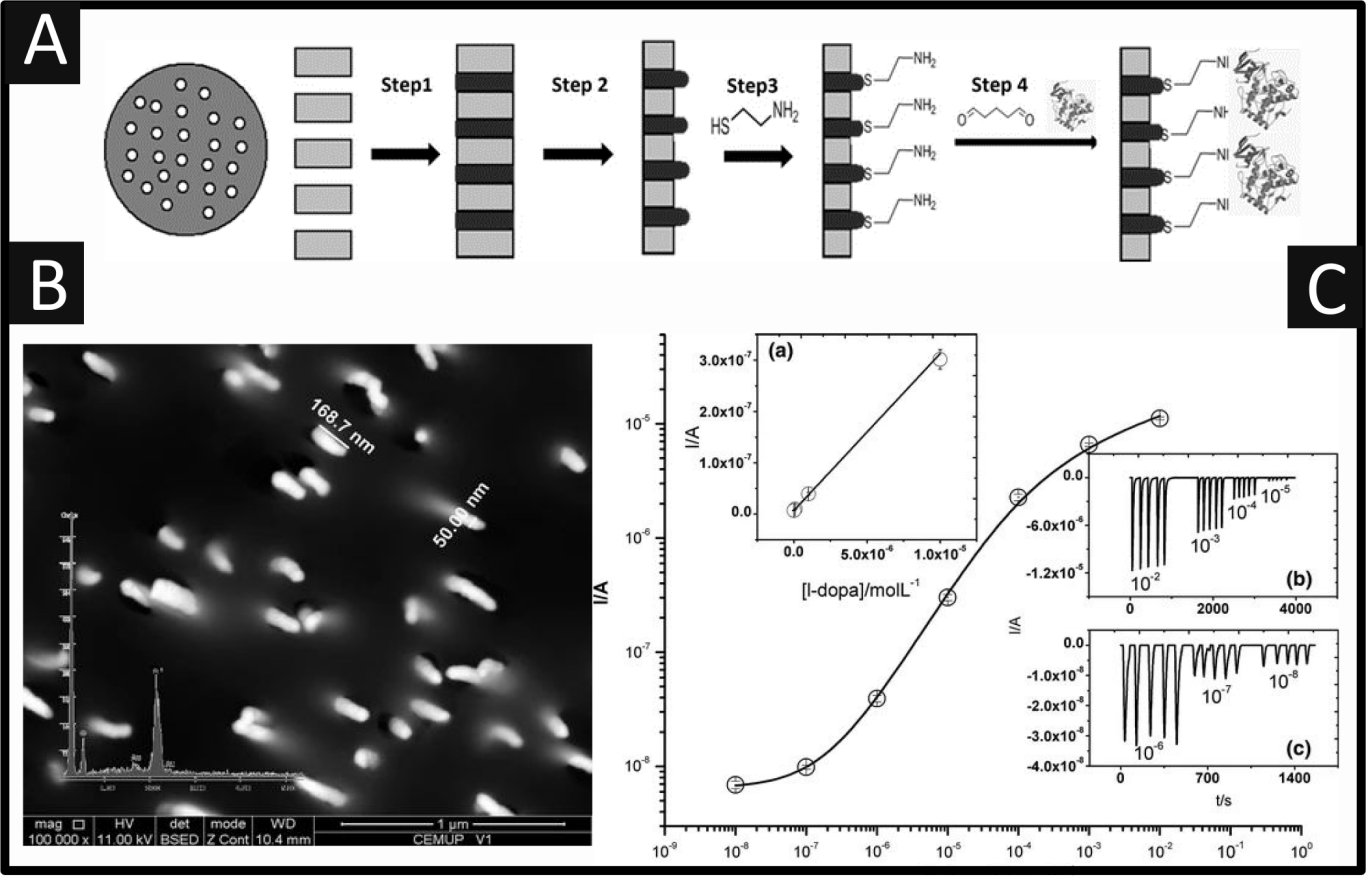
(A) Schematic representation of the biosensor’s (GNEE-Tyr) construction. Step 1, electroless gold deposition in PC membrane pores; step 2, partial etching and exposure of gold nanoarrays; step 3, self-assembled monolayer formation; step 4, tyrosinase immobilization by glutaraldehyde. (B) SEM image of 3D GNEEs. Inset, EDX spectrum of gold-filled PC membrane. (C) Dose–response curve for L-DOPA under optimized conditions. Inset a, linear fit of L-DOPA from 10^–8^ to 10^–5^ mol L^–1^; insets b and c, FIA responses for consecutive injections of L-DOPA solutions (10^–3^–10^–8^ mol L^–1^) in PBS (pH 6.5). Reproduced with permission from ref (49). Copyright 2012 Springer.

Pinho et al.^49^ reported the use of a 3D gold nanoelectrode ensemble (GNEE) in a flow-injection analysis system for the sensing of L-DOPA. A schematic representation of how the biosensor is prepared is shown in Figure 5A, which comprises of 4 steps involving the electroless gold deposition in polycarbonate membranes, followed by partial etching exposing gold nanoarrays. Figure 5B shows an SEM image of the 3D GNEE which shows that the gold nanowires have an average diameter of 50 nm and a length of 180 (±20) nm which also shows the absence of voids on surface, which suggest that this method produces 3D GNEEs with protruding gold wires.^49^ The 3D GNEE was explored toward the sensing of L-DOPA using a flow-injection analysis system, as shown in Figure 5C where a linear current response for L-DOPA between 10 nM and 10 mM was achieved. The LOD was found to be 1 nM with a resultant % RSD of 7.23% (*n* = 5). No significant interference from ascorbic acid, glucose, and urea on the measurement L-DOPA was observed. This approach was explored with human urine and had a recovery of 96%.

**Figure 6:**
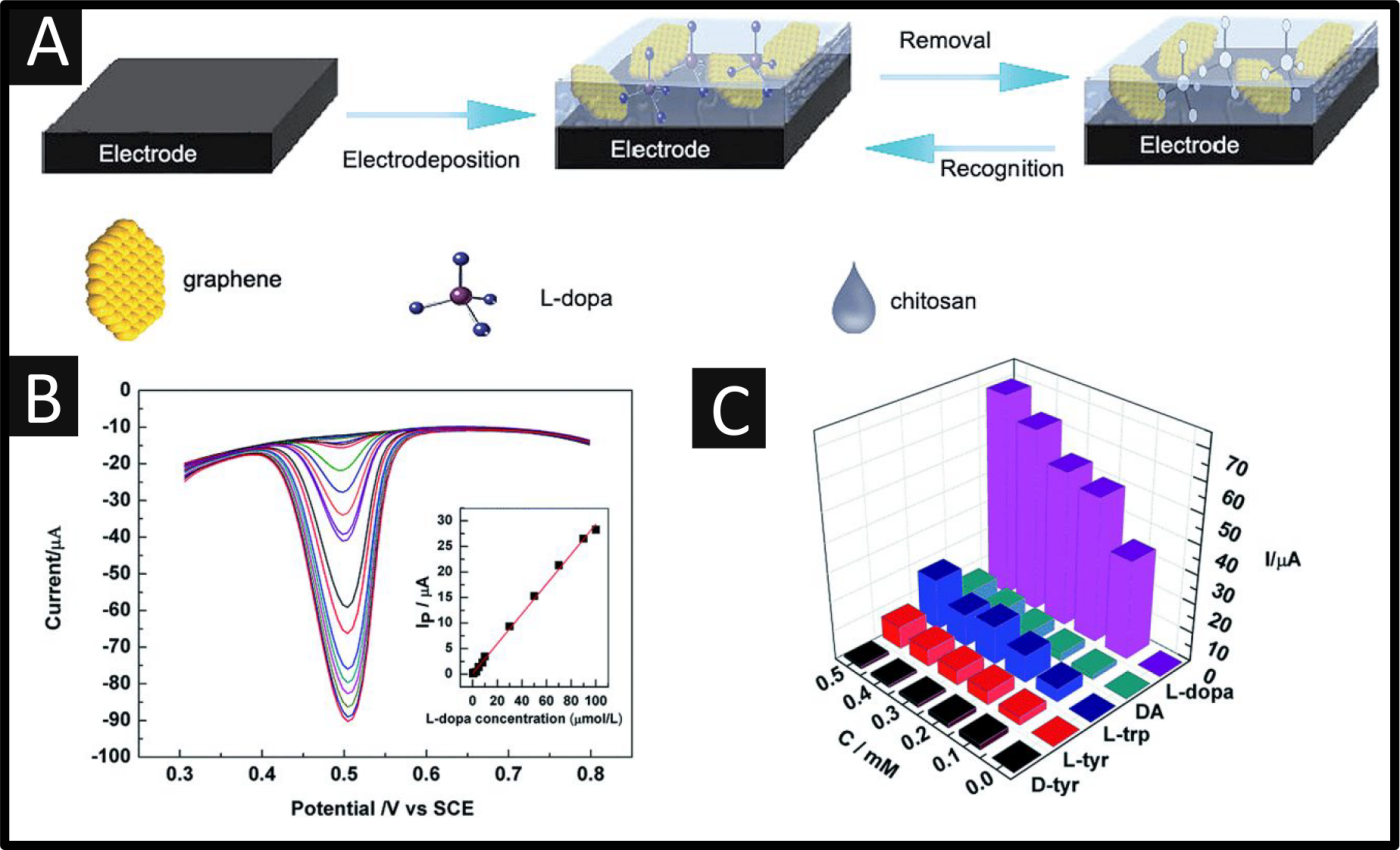
(A) Preparation of the graphene–MIP/GC and its recognition ability for L-DOPA. (B) DPV responses of the graphene–MIP/GC for L-DOPA from 0.4 μM to 100 μM. The inset shows the corresponding calibration plots with the concentration of L-DOPA ranging from 0.4 μM to 100 μM. (C) Responses of the graphene–MIP/GC for different structural analogues. Reproduced with permission from ref (50). Copyright 2015 The Royal Society of Chemistry.

Another approach is the design and development of Molecular Imprinted Polymers (MIPs).^38,50,63^ MIPs are synthetic receptors that can form high affinity binding sites corresponding to the specific analyte of interest^64^ and can utilize the shape, size, and functionality to produce sensitive and selective recognition of target analytes.^65^ Despite the advantages, there are few literature reports utilizing MIPs. Lin and co-workers have developed a selective sensor utilizing MIPs, based on a composite of graphene and chitosan.^50^Figure 6A shows how the graphene–MIP sensor was fabricated. The electrodeposition on a GC was achieved via holding the potential of −1.1 V for 150 s within a dispersion containing graphene, chitosan, and L-DOPA. The graphene–MIP/GCE was obtained after removing the template molecule from the composite by applying a potential of +0.6 V for 20 min within a new solution (0.1 M KCl solution containing 100 μL ethanol). A nonimprinted polymer sensor was prepared in the same way except that the template molecule was absent in the electrodeposition step. The sensor was evaluated for the sensing of L-DOPA which, as shown in Figure 6B, shows a linear response from 0.4 μM to 100 μM and a reported LOD of 12 nM. Figure 6C shows the specific recognition toward L-DOPA using the graphene–MIP/GCE sensor in the presence of d-tyrosine, l-tyrosine, l-tryptophan, and dopamine. The current in the presence of L-DOPA is the highest of all and changes significantly, which indicates that the sensor had a good specific recognition toward L-DOPA. The sensor was last evaluated for the measurement of L-DOPA within a pharmaceutical tablet and human blood serum. The authors conclude that their graphene–MIP/GC sensor exhibited a high sensitivity, low detection limit, good selectivity, and stability. In another example, following on from Lin and co-workers,^50^ Sooraj et al.^38^ developed copper nanoparticles (CuNPs) grafted with a MIP on MWCNTs. This was supported upon a GC electrode and was specific and selective giving the fabricated sensor a response to L-DOPA and not structurally related compounds such as dopamine, uric acid, 3,4-dihydroxyphenylacetic acid and homovanillic acid. This sensor was able to demonstrate a linear range of 0.01–1 μM and a LOD of 7.2 nM. This was extended to the measurement of L-DOPA within noninfected and infected human urine and pharmaceutical samples with a recovery between 98.3 and 102.4%.

As mentioned above, L-DOPA is administered with carbidopa for the treatment of Parkinson’s disease which prevents the conversion of L-DOPA into dopamine outside the brain, permitting more L-DOPA to reach the brain.^4^ L-DOPA is almost always given in combination with the drug carbidopa, which reduces or prevents the nausea that L-DOPA alone can cause. Reports of the simultaneous determination of L-DOPA in the presence of carbidopa are rather limited,^48,66,67^ despite the need for both in pharmaceuticals and human samples; this is an area that needs progressing.

**Figure 7:**
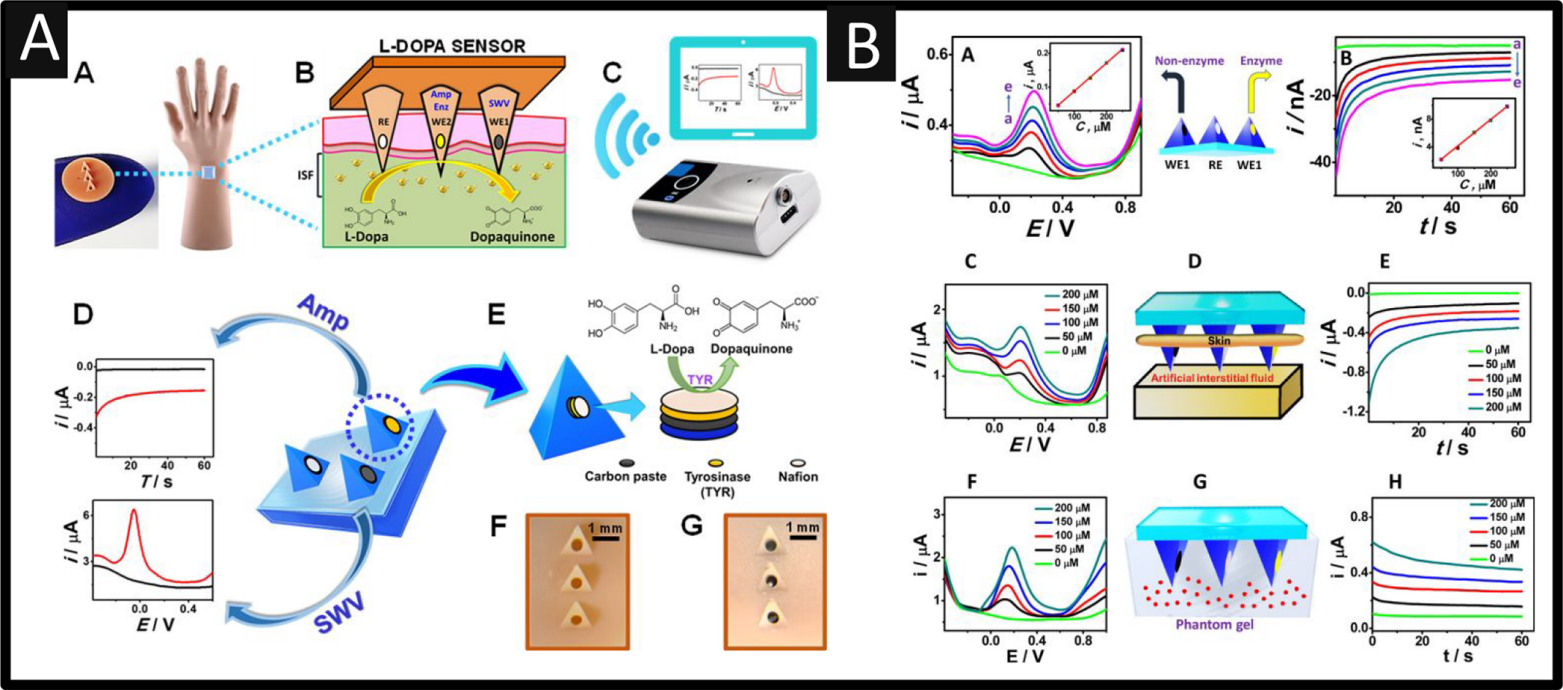
(A) Microneedle sensor for L-DOPA detection; schematic representation of (A) mannequin hand wearing the microneedle sensor and (B) the microneedle sensor for L-DOPA monitoring in ISF; (C) Portable wireless electroanalyzer enabled with wireless data transmission to the smart device; (D) schematics of the microneedle sensor platform, illustrating three electrodes used for L-DOPA sensing using SWV and amperometry; (E) microneedle sensor with the corresponding reagent layers, including the carbon paste, tyrosinase, and Nafion layer; (F, G) actual optical images of microneedles before and after packing with carbon paste (scale bar, 1 mm). (B) (A) Square-wave voltammograms for L-DOPA in ISF from 50 to 250 μM concentrations in 50 μM increments (inset shows the calibration plot of the background-subtracted peak-current vs L-DOPA concentration); (B) chronoamperometry responses of the L-DOPA biosensor recorded in ISF from 50 to 250 μM in 20 μM increments at 0.3 V vs Ag/AgCl electrode (inset shows the corresponding calibration plots); (C, E) real-time L-DOPA detection in artificial ISF through the mouse skin-penetrating microneedle; (F, H) SWV and amperometric responses of different L-DOPA concentrations with the mimicking-skin phantom gel; (D) schematic showing the detection of L-DOPA in artificial interstitial fluid using the microneedle penetrated through the mouse skin; (G) schematic representation of the skin-mimicking phantom gel with the penetration of the microneedle. Reprinted with permission from ref (5). Copyright 2019 American Chemical Society.

Wang and co-workers^5^ reported for the first time a fantastic microneedle sensing platform for continuous minimally invasive electrochemical sensing of L-DOPA. The motivation was an urgent need for a reliable sensing device to provide timely individualized feedback on the proper L-DOPA dosing regimen in a decentralized and rapid fashion.^5^Figure 7A shows an overview of their direct (nonenzymatic) L-DOPA sensing which was carried out using square-wave voltammetry and a microneedle, while a tyrosinase (TYR)-based biocatalytic detection was performed at a neighboring enzyme-paste microneedle electrode using chronoamperometric measurements of the corresponding dopaquinone product with the third microneedle completing the circuit as the reference electrode. The three-working electrode microneedle array was fabricated using carbon paste (CP) that was packed into the hollow microneedles. Figure 7B shows the square-wave voltammetry and chronoamperometry experiments that were performed simultaneously on three CP-modified microneedle electrodes (Figure 7B(A,B)). The square-wave voltammetry and the chronoamperometric response of both microneedle sensors were recorded over the range of 50 to 250 L-DOPA to artificial interstitial fluid. As shown in Figure 7B(C,E), the sensing of L-DOPA is achieved for artificial interstitial fluid through mouse skin, and the response of the sensor is shown for skin-mimicking phantom gel (Figure 7B(F,H)). Future work needs to focus on antibiofouling protective coatings and clinical testing and validation in Parkinson’s disease patients.

More recently, additively manufactured electrodes (AMEs) have been utilized for the production of electroanalytical platforms.^68^ In this way, AMEs of varying shapes and sizes^69^ can be 3D-printed and utilized in various systems, even with the electrodes printed within the cell walls.^68,70^ Whittingham et al.^71^ are the only report to date of an AME for the detection of L-DOPA, using it to show the proficiency of their 3D-printed rotating disc electrode and experimental setup. They report the use of additive manufacturing (AM) to produce both the rotator housing and disc electrode for their setup and compare this to a commercially purchased system with a GC rotating electrode. They show that L-DOPA could be detected with a LOD of 0.23 μM using an experimental set up of less than 2% and electrode of less than 0.05% material cost of the comparative commercial options. It should be noted that to produce the best AM electrochemical platforms, the connection length of AMEs should be kept to as short a distance as possible.^72^ Currently, these AMEs must be used as single-shot electrodes, similar to SPEs, because of solution ingress into the electrodes,^73^ among other issues. However, due to the flexibility, low-cost, and rapid prototyping capabilities of AM (in addition to the recent reports of the use of recycled conductive feedstocks^74^), we expect many further reports in this field over the coming years.

## Summary and Outlook

In our review, we have overviewed the electroanalytical sensing of L-DOPA, which provides credible approaches with high sensitivity and selectivity over common analytes that have the potential to be low-cost and portable. This is crucial due to the desire for rapid monitoring of L-DOPA dosages for the optimal treatment of Parkinson’s disease. One area that needs progressing is the measurement of L-DOPA and carbidopa within pharmaceuticals and human samples since these are both given to patients since it prevents the nausea that L-DOPA alone can cause. The measurement of L-DOPA in the literature is rather limited to pharmaceuticals and blood (serum) and urine, and only a handful have measured L-DOPA within food. Note that it is reported that the global demand for L-DOPA has an annual market value of USD 2.64 billion in 2020.^75^ For example, it is well-known that relatively high L-DOPA concentrations occur in natural sources, such as *Mucuna* and fava beans, which have been measured using LC-MS,^76^ and other plant species,^77^ which could lead to a dietary supplement; this is an area that could be beneficial for electroanalytical sensors.

## Abbreviations:

AME: additively manufactured electrode
ASV: adsorption stripping voltammetry
AuNP: gold nanoparticle
BDD: boron-doped diamond
CAc: cellulose acetate
CILE: carbon ionic liquid electrode
CMC: carboxymethylcelullose
CuNP: copper nanoparticle
CPE: carbon paste electrode
CZE: capillary zone electrophoresis
DE: 1-(6,7-dihydroxy-2,4-dimethylbenzofuran-3-yl) ethenone
DPV: differential pulse voltammetry
EBNBH: 2,2′-[1,2-ethanediylbis(nitriloethylidyne)]-bis-hydroquinone
EFTA: ethyl 2-(4-ferrocenyl-[1,2,3]triazol-1-yl) acetate
gold-SPE: gold screen-printed electrode
GPE: graphite–polyurethane electrode
fMWCNT: functionalized multiwall carbon nanotube
GNEE: 3D gold nanoelectrode ensemble
GCE: glassy carbon electrode
GE: graphite electrode
GCPE: glassy carbon paste electrode
GF: graphene foam
GP: graphite powder
GPE: graphite–polyurethane electrode
GnP: exfoliated graphite nanoplatelet
Hb: hemoglobin
HRP/MWCNT-pPDA: horseradish peroxidase, multiwalled carbon nanotube–p-phenylenediamine glassy carbon
ITO: indium tin oxide glass
LMCGC: large-mesopore carbon modified glassy carbon electrode
MIP: molecular imprinted polymer
MOF: metal–organic framework
NaY: NaY zeolite
p-Ni^II^TAPc: 3,3′,3″,3‴-tetraaminophthalocyanatonickel(II)
NiNP: nickel nanoparticle
NPSS: nanoporous stainless steel
N-GE/NiO: nitrogen-doped graphene supported with nickel oxide
PEDOT: poly(3,4-)ethylenedioxythiophene
PPy: polypyrrole
PEG: poly(ethylene glycol)
PCFCuNP: polycysteine functionalized copper nanoparticle
PVC: poly(vinyl chloride)
IL: ionic liquid
PE: paste electrode
PGE: pencil graphite electrode
RDE: rotating disc electrode
RGO: reduced graphene oxide
Ru-red: [(NH_3_)_5_Ru^III^-O-Ru^IV^(NH_3_)_4_-O-Ru^III^(NH_3_)_5_]^6+^
SWV: square-wave voltammetry
SPE: screen-printed electrodes
WO_3_NP: tungsten oxide nanoparticle
ZnONF: zinc oxide nanofiber
ZnO NR: zinc oxide nanorod
3D HGB: 3-dimensional hollow graphene ball
35DT: 3,5-diamino-1,2,4-triazole
3,4′-AAZ: 3-(4′-amino-3′-hydroxy-biphenyl-4-yl)-acrylic acid

## References

[ref1] Hornykiewicz O. L-DOPA: From a biologically inactive amino acid to a successful therapeutic agent. Amino Acids 2002, 23 (1), 65–70. 10.1007/s00726-001-0111-9. 12373520

[ref2] De Deurwaerdère P.; Di Giovanni G.; Millan M. J. Expanding the repertoire of L-DOPA’s actions: A comprehensive review of its functional neurochemistry. Progress in neurobiology 2017, 151, 57–100. 10.1016/j.pneurobio.2016.07.002. 27389773

[ref3] https://www.age-platform.eu/sites/default/files/EPDA-Political_Manifesto_Parkinson.pdf. (accessed 2023 Jan).

[ref4] Gandhi K. R.; Saadabadi A. Levodopa (L-Dopa).StatPearls [Internet]. StatPearls Publishing, Treasure Island (FL); 2022 Jan. [Updated 2022 May 2] Available from: https://www.ncbi.nlm.nih.gov/books/NBK482140/.

[ref5] Goud K. Y.; Moonla C.; Mishra R. K.; Yu C.; Narayan R.; Litvan I.; Wang J. Wearable Electrochemical Microneedle Sensor for Continuous Monitoring of Levodopa: Toward Parkinson Management. ACS Sensors 2019, 4 (8), 2196–2204. 10.1021/acssensors.9b01127. 31403773

[ref6] Albin R. L.; Leventhal D. K. The missing, the short, and the long: Levodopa responses and dopamine actions. Ann. Neurol. 2017, 82, 4–19. 10.1002/ana.24961. 28543679 PMC5526730

[ref7] Elbarbry F.; Nguyen V.; Mirka A.; Zwickey H.; Rosenbaum R. A new validated HPLC method for the determination of levodopa: Application to study the impact of ketogenic diet on the pharmacokinetics of levodopa in Parkinson’s participants. Biomed Chromatogr 2019, 33 (1), e4382 10.1002/bmc.4382. 30203852

[ref8] Loutelier-Bourhis C.; Legros H.; Bonnet J. J.; Costentin J.; Lange C. M. Gas chromatography/mass spectrometric identification of dopaminergic metabolites in striata of rats treated with L-DOPA. Rapid Commun. Mass Spectrom. 2004, 18 (5), 571–576. 10.1002/rcm.1369. 14978802

[ref9] César I. C.; Byrro R. M. D.; de Santana e Silva Cardoso F. F.; Mundim I. M.; de Souza Teixeira L.; Gomes S. A.; Bonfim R. R.; Pianetti G. A. Development and validation of a high-performance liquid chromatography–electrospray ionization–MS/MS method for the simultaneous quantitation of levodopa and carbidopa in human plasma. Journal of Mass Spectrometry 2011, 46 (9), 943–948. 10.1002/jms.1973. 21915959

[ref10] García-Miranda Ferrari A.; Rowley-Neale S. J.; Banks C. E. Screen-printed electrodes: Transitioning the laboratory in-to-the field. Talanta Open 2021, 3, 100032 10.1016/j.talo.2021.100032.

[ref11] Ferrari A. G.-M.; Crapnell R. D.; Banks C. E. Electroanalytical Overview: Electrochemical Sensing Platforms for Food and Drink Safety. Biosensors 2021, 11, 291 10.3390/bios11080291. 34436093 PMC8392528

[ref12] Blandini F.; Martignoni E.; Pacchetti C.; Desideri S.; Rivellini D.; Nappi G. Simultaneous determination of l-dopa and 3-O-methyldopa in human platelets and plasma using high-performance liquid chromatography with electrochemical detection. Journal of Chromatography B: Biomedical Sciences and Applications 1997, 700 (1), 278–282. 10.1016/S0378-4347(97)00307-1. 9390741

[ref13] Rizzo V.; Pastore R.; Pankopf S.; Melzi; D’Eril G. V.; Moratti R. Determination of total and non-protein-bound fractions of L-Dopa in blood plasma by liquid chromatography and electro-chemistry. Biogenic Amines 1996, 12 (1), 1–7.

[ref14] Galal A.; Atta N. F.; Rubinson J. F.; Zimmer H.; Mark H. B. Electrochemistry and Detection of Some Organic and Biological Molecules at Conducting Polymer Electrodes. II. Effect of Nature of Polymer Electrode and Substrate on Electrochemical Behavior and Detection of Some Neurotransmitters. Anal. Lett. 1993, 26 (7), 1361–1381. 10.1080/00032719308017418.

[ref15] Dutton J.; Copeland L. G.; Playfer J. R.; Roberts N. B. Measuring L-dopa in plasma and urine to monitor therapy of elderly patients with Parkinson disease treated with L-dopa and a dopa decarboxylase inhibitor. Clin Chem. 1993, 39 (4), 629–634. 10.1093/clinchem/39.4.629. 8472357

[ref16] Sagar K. A.; Smyth M. R. Simultaneous determination of levodopa, carbidopa and their metabolites in human plasma and urine samples using LC-EC. J. Pharm. Biomed. Anal. 2000, 22 (3), 613–624. 10.1016/S0731-7085(00)00237-5. 10766378

[ref17] Stanković D. M.; Samphao A.; Dojcinović B.; Kalcher K. Rapid electrochemical method for the determination of L-DOPA in extract from the seeds of Mucuna prurita. Acta Chim. Slov. 2016, 63 (2), 220 10.17344/acsi.2015.1541. 27333543

[ref18] Wang J.; Zhou Y.; Liang J.; He P. G.; Fang Y. Z. Determination of Levodopa and Benserazide Hydrochloride in Pharmaceutical Formulations by CZE with Amperometric Detection. Chromatographia 2005, 61 (5), 265–270. 10.1365/s10337-005-0515-x.

[ref19] Mascarenhas R. J.; Reddy K. V.; Kumaraswamy B. E.; Sherigara B. S.; Lakshminarayanan V. Electrochemical oxidation of L-dopa at a carbon paste electrode. Bulletin of Electrochemistry 2005, 8, 341–345.

[ref20] Xiang C.; Zou Y.; Xie J.; Fei X. Voltammetric Determination of L-Dopa Using a Carbon Nanotubes-Nafion Modified Glassy Carbon Electrode. Anal. Lett. 2006, 39 (13), 2569–2579. 10.1080/00032710600824706.

[ref21] Beitollahi H.; Raoof J.-B.; Hosseinzadeh R. Application of a Carbon-Paste Electrode Modified with 2,7-Bis(ferrocenyl ethyl)fluoren-9-one and Carbon Nanotubes for Voltammetric Determination of Levodopa in the Presence of Uric Acid and Folic Acid. Electroanalysis 2011, 23 (8), 1934–1940. 10.1002/elan.201100242.

[ref22] Shoja Y.; Rafati A. A.; Ghodsi J. Glassy carbon electrode modified with horse radish peroxidase/organic nucleophilic-functionalized carbon nanotube composite for enhanced electrocatalytic oxidation and efficient voltammetric sensing of levodopa. Materials Science and Engineering: C 2016, 58, 835–845. 10.1016/j.msec.2015.09.028. 26478378

[ref23] Raoof J. B.; Ojani R.; Amiri-Aref M.; Baghayeri M. Electrodeposition of quercetin at a multi-walled carbon nanotubes modified glassy carbon electrode as a novel and efficient voltammetric sensor for simultaneous determination of levodopa, uric acid and tyramine. Sens. Actuators, B 2012, 166–167, 508–518. 10.1016/j.snb.2012.02.096.

[ref24] Ghodsi J.; Rafati A. A.; Shoja Y. First report on hemoglobin electrostatic immobilization on WO3 nanoparticles: application in the simultaneous determination of levodopa, uric acid, and folic acid. Anal. Bioanal. Chem. 2016, 408 (14), 3899–3909. 10.1007/s00216-016-9480-5. 27007733

[ref25] Baião V.; Tomé L. I. N.; Brett C. M. A. Iron Oxide Nanoparticle and Multiwalled Carbon Nanotube Modified Glassy Carbon Electrodes. Application to Levodopa Detection. Electroanalysis 2018, 30 (7), 1342–1348. 10.1002/elan.201700854.

[ref26] Akhgar M. R.; Salari M.; Zamani H. Simultaneous determination of levodopa, NADH, and tryptophan using carbon paste electrode modified with carbon nanotubes and ferrocenedicarboxylic acid. J. Solid State Electrochem. 2011, 15 (4), 845–853. 10.1007/s10008-010-1158-x.

[ref27] Hu G.; Chen L.; Guo Y.; Wang X.; Shao S. Selective determination of L-dopa in the presence of uric acid and ascorbic acid at a gold nanoparticle self-assembled carbon nanotube-modified pyrolytic graphite electrode. Electrochim. Acta 2010, 55 (16), 4711–4716. 10.1016/j.electacta.2010.03.069.

[ref28] Yan X.-X.; Pang D.-W.; Lu Z.-X.; Lü J.-Q.; Tong H. Electrochemical behavior of l-dopa at single-wall carbon nanotube-modified glassy carbon electrodes. J. Electroanal. Chem. 2004, 569 (1), 47–52. 10.1016/j.jelechem.2004.02.011.

[ref29] Babaei A.; Taheri A. R.; Aminikhah M. Nanomolar simultaneous determination of levodopa and serotonin at a novel carbon ionic liquid electrode modified with Co(OH)2 nanoparticles and multi-walled carbon nanotubes. Electrochim. Acta 2013, 90, 317–325. 10.1016/j.electacta.2012.11.121.

[ref30] Babaei A.; Babazadeh M. A Selective Simultaneous Determination of Levodopa and Serotonin Using a Glassy Carbon Electrode Modified with Multiwalled Carbon Nanotube/Chitosan Composite. Electroanalysis 2011, 23 (7), 1726–1735. 10.1002/elan.201000755.

[ref31] Shaikshavali P.; Reddy T. M.; Venkataprasad G.; Gopal P. A highly selective electrochemical sensor based on multi walled carbon nano tubes/poly (Evans blue) composite for the determination of l-dopa in presence of 5-HT and folic acid: a voltammetric investigation. Journal of the Iranian Chemical Society 2018, 15 (8), 1831–1841. 10.1007/s13738-018-1380-5.

[ref32] Brunetti B.; Valdés-Ramírez G.; Litvan I.; Wang J. A disposable electrochemical biosensor for l-DOPA determination in undiluted human serum. Electrochem. Commun. 2014, 48, 28–31. 10.1016/j.elecom.2014.08.007.

[ref33] Nasirizadeh N.; Shekari Z.; Tabatabaee M.; Ghaani M. Simultaneous Determination of Ascorbic Acid, L-Dopa, Uric Acid, Insulin, and Acetylsalicylic Acid on Reactive Blue 19 and Multi-Wall Carbon Nanotube Modified Glassy Carbon Electrode. J. Braz. Chem. Soc. 2015, 26 (4), 713–722. 10.5935/0103-5053.20150031.

[ref34] Afkhami A.; Kafrashi F.; Madrakian T. Electrochemical determination of levodopa in the presence of ascorbic acid by polyglycine/ZnO nanoparticles/multi-walled carbon nanotubes-modified carbon paste electrode. Ionics 2015, 21 (10), 2937–2947. 10.1007/s11581-015-1486-z.

[ref35] Mathiyarasu J.; Nyholm L. Voltammetric Determination of L-Dopa on Poly(3,4-ethylenedioxythiophene)-Single-Walled Carbon Nanotube Composite Modified Microelectrodes. Electroanalysis 2010, 22 (4), 449–454. 10.1002/elan.200900340.

[ref36] Đurđić S.; Stanković V.; Vlahović F.; Ognjanović M.; Kalcher K.; Manojlović D.; Mutić J.; Stanković D. M. Carboxylated single-wall carbon nanotubes decorated with SiO2 coated-Nd2O3 nanoparticles as an electrochemical sensor for L-DOPA detection. Microchemical Journal 2021, 168, 106416 10.1016/j.microc.2021.106416.

[ref37] Tu Y.; Xu Q.; Zou Q. J.; Yin Z. H.; Sun Y. Y.; Zhao Y. D. Electrochemical behavior of levodopa at multi-wall carbon nanotubes-quantum dots modified glassy carbon electrodes. Anal. Sci. 2007, 23 (11), 1321–1324. 10.2116/analsci.23.1321. 17998753

[ref38] Sooraj M. P.; Nair A. S.; Pillai S. C.; Hinder S. J.; Mathew B. CuNPs decorated molecular imprinted polymer on MWCNT for the electrochemical detection of l-DOPA. Arabian Journal of Chemistry 2020, 13 (1), 2483–2495. 10.1016/j.arabjc.2018.06.002.

[ref39] Beitollahi H.; Taher M. A.; Keshtkar N. Voltammetric Determination of L-Dopa in the Presence of Tryptophan Using a Modified Carbon Nanotubes Paste Electrode. Sensor Letters 2014, 12 (1), 183–190. 10.1166/sl.2014.3230.

[ref40] Babaei A.; Babazadeh M.; Afrasiabi M. A Sensitive Simultaneous Determination of L-Dopa and Acetaminophen on a Glassy Carbon Electrode Modifed with a Film of SWCNT-CHIT-IL Nanocomposite. Sensor Letters 2012, 10 (3–4), 993–999. 10.1166/sl.2012.2327.

[ref41] Mazloum-Ardakani M.; Ganjipour B.; Beitollahi H.; Amini M. K.; Mirkhalaf F.; Naeimi H.; Nejati-Barzoki M. Simultaneous determination of levodopa, carbidopa and tryptophan using nanostructured electrochemical sensor based on novel hydroquinone and carbon nanotubes: Application to the analysis of some real samples. Electrochim. Acta 2011, 56 (25), 9113–9120. 10.1016/j.electacta.2011.07.021.

[ref42] Iijima S. Helical microtubules of graphitic carbon. Nature 1991, 354 (6348), 56–58. 10.1038/354056a0.

[ref43] Kaliyaraj Selva Kumar A.; Lu Y.; Compton R. G. Voltammetry of Carbon Nanotubes and the Limitations of Particle-Modified Electrodes: Are Carbon Nanotubes Electrocatalytic. J. Phys. Chem. Lett. 2022, 13 (37), 8699–8710. 10.1021/acs.jpclett.2c02464. 36094419 PMC9511562

[ref44] Yue H. Y.; Song S. S.; Huang S.; Zhang H.; Gao X. P. A.; Gao X.; Lin X. Y.; Yao L. H.; Guan E. H.; Zhang H. J. Preparation of MoS2-graphene Hybrid Nanosheets and Simultaneously Electrochemical Determination of Levodopa and Uric Acid. Electroanalysis 2017, 29 (11), 2565–2571. 10.1002/elan.201700329.

[ref45] Naghian E.; Shahdost-fard F.; Sohouli E.; Safarifard V.; Najafi M.; Rahimi-Nasrabadi M.; Sobhani-Nasab A. Electrochemical determination of levodopa on a reduced graphene oxide paste electrode modified with a metal-organic framework. Microchemical Journal 2020, 156, 104888 10.1016/j.microc.2020.104888.

[ref46] Martín A.; Hernández-Ferrer J.; Martínez M. T.; Escarpa A. Graphene nanoribbon-based electrochemical sensors on screen-printed platforms. Electrochim. Acta 2015, 172, 2–6. 10.1016/j.electacta.2014.11.090.

[ref47] Renganathan V.; Sasikumar R.; Chen S.-M.; Chen T.-W.; Rwei S.-P.; Lee S.-Y.; Chang W.-H.; Lou B.-S. Detection of Neurotransmitter (Levodopa) in Vegetables Using Nitrogen-Doped Graphene Oxide Incorporated Nickel Oxide Modified Electrode. Int. J. Electrochem. Sci. 2018, 13, 7206–7217. 10.20964/2018.07.700.

[ref48] Wang Q.; Das M. R.; Li M.; Boukherroub R.; Szunerits S. Voltammetric detection of l-dopa and carbidopa on graphene modified glassy carbon interfaces. Bioelectrochemistry 2013, 93, 15–22. 10.1016/j.bioelechem.2012.03.004. 22513265

[ref49] Pinho A.; Viswanathan S.; Ribeiro S.; Oliveira M. B. P. P.; Delerue-Matos C. Electroanalysis of urinary l-dopa using tyrosinase immobilized on gold nanoelectrode ensembles. J. Appl. Electrochem. 2012, 42 (3), 131–137. 10.1007/s10800-012-0379-3.

[ref50] Lin L.; Lian H.-T.; Sun X.-Y.; Yu Y.-M.; Liu B. An l-dopa electrochemical sensor based on a graphene doped molecularly imprinted chitosan film. Analytical Methods 2015, 7 (4), 1387–1394. 10.1039/C4AY02524E.

[ref51] Geim A. K.; Novoselov K. S. The rise of graphene. Nat. Mater. 2007, 6 (3), 183–191. 10.1038/nmat1849. 17330084

[ref52] Arvand M.; Ghodsi N. A voltammetric sensor based on graphene-modified electrode for the determination of trace amounts of l-dopa in mouse brain extract and pharmaceuticals. J. Solid State Electrochem. 2013, 17 (3), 775–784. 10.1007/s10008-012-1929-7.

[ref53] Brownson D. A. C.; Munro L. J.; Kampouris D. K.; Banks C. E. Electrochemistry of graphene: not such a beneficial electrode material. RSC Adv. 2011, 1 (6), 978–988. 10.1039/c1ra00393c.

[ref54] Brownson D. A.; Kampouris D. K.; Banks C. E. Graphene electrochemistry: fundamental concepts through to prominent applications. Chem. Soc. Rev. 2012, 41 (21), 6944–6976. 10.1039/c2cs35105f. 22850696

[ref55] Liu X.; Zhang Z.; Cheng G.; Dong S. Spectroelectrochemical and Voltammetric Studies of L-DOPA. Electroanalysis 2003, 15 (2), 103–107. 10.1002/elan.200390009.

[ref56] Crapnell R. D.; Banks C. E. Electroanalytical overview: utilising micro-and nano-dimensional sized materials in electrochemical-based biosensing platforms. Microchimica Acta 2021, 188 (8), 268 10.1007/s00604-021-04913-y. 34296349 PMC8298255

[ref57] Ji X.; Kadara R. O.; Krussma J.; Chen Q.; Banks C. E. Understanding the Physicoelectrochemical Properties of Carbon Nanotubes: Current State of the Art. Electroanalysis 2010, 22 (1), 7–19. 10.1002/elan.200900493.

[ref58] Bergamini M. F.; Santos A. L.; Stradiotto N. R.; Zanoni M. V. B. A disposable electrochemical sensor for the rapid determination of levodopa. J. Pharm. Biomed. Anal. 2005, 39 (1), 54–59. 10.1016/j.jpba.2005.03.014. 15896939

[ref59] García-Miranda Ferrari A.; Carrington P.; Rowley-Neale S. J.; Banks C. E. Recent advances in portable heavy metal electrochemical sensing platforms. Environmental Science: Water Research & Technology 2020, 6 (10), 2676–2690. 10.1039/D0EW00407C.

[ref60] Crapnell R.; Ferrari A. G.-M.; Dempsey N.; Banks C. E. Electroanalytical Overview: Screen-printed electrochemical sensing platforms for the detection of vital cardiac, cancer and inflammatory biomarkers. Sensors & Diagnostics 2022, 1, 405–428. 10.1039/D1SD00041A.

[ref61] Noguchi H. K.; Kaur S.; Krettli L. M.; Singla P.; McClements J.; Snyder H.; Crapnell R. D.; Banks C. E.; Novakovic K.; Kaur I.; et al. Rapid electrochemical detection of levodopa using polyaniline-modified screen-printed electrodes for the improved management of Parkinson’s disease. Physics in Medicine 2022, 14, 100052 10.1016/j.phmed.2022.100052.

[ref62] Rabinca A. A.; Buleandra M.; Tache F.; Mihailciuc C.; Ciobanu A. M.; Stefanescu D. C.; Ciucu A. A. Voltammetric Method for Simultaneous Determination of L-Dopa and Benserazide. Current Analytical Chemistry 2017, 13 (3), 218–224. 10.2174/1573411012666160601161703.

[ref63] Trotta F.; Caldera F.; Cavalli R.; Soster M.; Riedo C.; Biasizzo M.; Uccello Barretta G.; Balzano F.; Brunella V. Molecularly imprinted cyclodextrin nanosponges for the controlled delivery of L-DOPA: perspectives for the treatment of Parkinson’s disease. Expert Opinion on Drug Delivery 2016, 13 (12), 1671–1680. 10.1080/17425247.2017.1248398. 27737572

[ref64] Crapnell R. D.; Dempsey-Hibbert N. C.; Peeters M.; Tridente A.; Banks C. E. Molecularly imprinted polymer based electrochemical biosensors: Overcoming the challenges of detecting vital biomarkers and speeding up diagnosis. Talanta Open 2020, 2, 100018 10.1016/j.talo.2020.100018.

[ref65] Crapnell R. D.; Hudson A.; Foster C. W.; Eersels K.; Grinsven Bv; Cleij T. J.; Banks C. E.; Peeters M. Recent Advances in Electrosynthesized Molecularly Imprinted Polymer Sensing Platforms for Bioanalyte Detection. Sensors 2019, 19, 1204 10.3390/s19051204. 30857285 PMC6427210

[ref66] Mazloum-Ardakani M.; Taleat Z.; Khoshroo A.; Beitollahi H.; Dehghani H. Electrocatalytic oxidation and voltammetric determination of levodopa in the presence of carbidopa at the surface of a nanostructure based electrochemical sensor. Biosens. Bioelectron. 2012, 35 (1), 75–81. 10.1016/j.bios.2012.02.014. 22410486

[ref67] Molaakbari E.; Mostafavi A.; Beitollahi H.; Alizadeh R. Synthesis of ZnO nanorods and their application in the construction of a nanostructure-based electrochemical sensor for determination of levodopa in the presence of carbidopa. Analyst 2014, 139 (17), 4356–4364. 10.1039/C4AN00138A. 25014312

[ref68] Whittingham M. J.; Crapnell R. D.; Rothwell E. J.; Hurst N. J.; Banks C. E. Additive manufacturing for electrochemical labs: an overview and tutorial note on the production of cells, electrodes and accessories. Talanta Open 2021, 4, 100051 10.1016/j.talo.2021.100051.

[ref69] Garcia-Miranda Ferrari A.; Hurst N. J.; Bernalte E.; Crapnell R. D.; Whittingham M. J.; Brownson D. A. C.; Banks C. E. Exploration of defined 2-dimensional working electrode shapes through additive manufacturing. Analyst 2022, 147 (22), 5121–5129. 10.1039/D2AN01412B. 36222111

[ref70] Shergill R. S.; Farlow A.; Perez F.; Patel B. A. 3D-printed electrochemical pestle and mortar for identification of falsified pharmaceutical tablets. Microchimica Acta 2022, 189 (3), 100 10.1007/s00604-022-05202-y. 35152330

[ref71] Whittingham M. J.; Crapnell R. D.; Banks C. E. Additively manufactured rotating disk electrodes and experimental setup. Analytical chemistry 2022, 94 (39), 13540–13548. 10.1021/acs.analchem.2c02884. 36129134 PMC9535625

[ref72] Crapnell R. D.; Garcia-Miranda Ferrari A.; Whittingham M. J.; Sigley E.; Hurst N. J.; Keefe E. M.; Banks C. E. Adjusting the Connection Length of Additively Manufactured Electrodes Changes the Electrochemical and Electroanalytical Performance. Sensors 2022, 22 (23), 9521 10.3390/s22239521. 36502222 PMC9736051

[ref73] Williams R. J.; Brine T.; Crapnell R. D.; Ferrari A. G.-M.; Banks C. E. The effect of water ingress on additively manufactured electrodes. Materials Advances 2022, 3 (20), 7632–7639. 10.1039/D2MA00707J.

[ref74] Wuamprakhon P.; Crapnell R. D.; Sigley E.; Hurst N. J.; Williams R. J.; Sawangphruk M.; Keefe E. M.; Banks C. E. Recycled Additive Manufacturing Feedstocks for Fabricating High Voltage, Low-Cost Aqueous Supercapacitors. Advanced Sustainable Systems 2022, 2200407 10.1002/adsu.202200407.

[ref75] Parkinson’s Disease Therapeutics. Market Size By Drug Class (Levodopa/Carbidopa, Dopamine Agonists, Adenosine A2A Antagonist, COMT Inhibitors, MAO-B Inhibitors, Glutamate Antagonist), By Route of Administration (Oral, Subcutaneous, Transdermal), By Patient (Adult, Pediatric), COVID-19 Impact Analysis, Regional Outlook, Application Potential, Competitive Market Share & Forecast, 2021 – 2027. Global Market Insights, 2021.

[ref76] Yumoto E.; Yanagihara N.; Asahina M. The simple and rapid quantification method for L-3,4-dihydroxyphenylalanine (L-DOPA) from plant sprout using liquid chromatography-mass spectrometry. Plant Biotechnol (Tokyo) 2022, 39 (2), 199–204. 10.5511/plantbiotechnology.21.1126a. 35937524 PMC9300427

[ref77] Tesoro C.; Lelario F.; Ciriello R.; Bianco G.; Di Capua A.; Acquavia M. A. An Overview of Methods for L-Dopa Extraction and Analytical Determination in Plant Matrices. Separations 2022, 9, 224 10.3390/separations9080224.

[ref78] Sivanesan A.; John S. A. Determination of l-dopa using electropolymerized 3,3′,3″,3‴-tetraaminophthalocyanatonickel(II) film on glassy carbon electrode. Biosens. Bioelectron. 2007, 23 (5), 708–713. 10.1016/j.bios.2007.08.005. 17888649

[ref79] Yan X.; Pan D.; Wang H.; Bo X.; Guo L. Electrochemical determination of L-dopa at cobalt hexacyanoferrate/large-mesopore carbon composite modified electrode. J. Electroanal. Chem. 2011, 663 (1), 36–42. 10.1016/j.jelechem.2011.09.024.

[ref80] Prabhu P.; Suresh Babu R.; Sriman Narayanan S. Amperometric determination of l-dopa by nickel hexacyanoferrate film modified gold nanoparticle graphite composite electrode. Sens. Actuators, B 2011, 156 (2), 606–614. 10.1016/j.snb.2011.02.006.

[ref81] Kannan A.; Radhakrishnan S. Fabrication of an electrochemical sensor based on gold nanoparticles functionalized polypyrrole nanotubes for the highly sensitive detection of l-dopa. Materials Today Communications 2020, 25, 101330 10.1016/j.mtcomm.2020.101330.

[ref82] Hariharan V.; Radhakrishnan S.; Parthibavarman M.; Dhilipkumar R.; Sekar C. Synthesis of polyethylene glycol (PEG) assisted tungsten oxide (WO3) nanoparticles for l-dopa bio-sensing applications. Talanta 2011, 85 (4), 2166–2174. 10.1016/j.talanta.2011.07.063. 21872074

[ref83] Aslanoglu M.; Kutluay A.; Goktas S.; Karabulut S. Voltammetric behaviour of levodopa and its quantification in pharmaceuticals using a β-cyclodextrine doped poly (2,5-diaminobenzenesulfonic acid) modified electrode. Journal of Chemical Sciences 2009, 121 (2), 209–215. 10.1007/s12039-009-0024-9.

[ref84] Teixeira M. F. S.; Bergamini M. F.; Marques C. M. P.; Bocchi N. Voltammetric determination of L-dopa using an electrode modified with trinuclear ruthenium ammine complex (Ru-red) supported on Y-type zeolite. Talanta 2004, 63 (4), 1083–1088. 10.1016/j.talanta.2004.01.018. 18969537

[ref85] Teixeira M. F. S.; Marcolino-Júnior L. H.; Fatibello-Filho O.; Dockal E. R.; Bergamini M. F. An electrochemical sensor for l-dopa based on oxovanadium-salen thin film electrode applied flow injection system. Sens. Actuators, B 2007, 122 (2), 549–555. 10.1016/j.snb.2006.06.032.

[ref86] Yue H. Y.; Wang B.; Huang S.; Gao X.; Song S. S.; Guan E. H.; Zhang H. J.; Wu P. F.; Guo X. R. Synthesis of graphene/ZnO nanoflowers and electrochemical determination of levodopa in the presence of uric acid. Journal of Materials Science: Materials in Electronics 2018, 29 (17), 14918–14926. 10.1007/s10854-018-9630-y.

[ref87] Gao X.; Yue H.; Song S.; Huang S.; Li B.; Lin X.; Guo E.; Wang B.; Guan E.; Zhang H.; et al. 3-Dimensional hollow graphene balls for voltammetric sensing of levodopa in the presence of uric acid. Microchimica Acta 2018, 185 (2), 91 10.1007/s00604-017-2644-y. 29594616

[ref88] Zhang J.; Wang Q.; Sun Z.; Buhe B.; He X. Fabrication the hybrization of ZnO nanorods–Graphene nanoslices and their electrochemical properties to Levodopa in the presence of uric acid. Journal of Materials Science: Materials in Electronics 2018, 29 (19), 16894–16902. 10.1007/s10854-018-9784-7.

[ref89] Ensafi A. A.; Arabzadeh A. A.; Karimi-Maleh H. Sequential determination of benserazide and levodopa by voltammetric method using chloranil as a mediator. J. Braz. Chem. Soc. 2010, 21, 1572–1580. 10.1590/S0103-50532010000800024.

[ref90] Yue H. Y.; Zhang H.; Huang S.; Gao X.; Chang J.; Lin X. Y.; Yao L. H.; Wang L. P.; Guo E. J. Selective determination of L-dopa in the presence of ascorbic acid and uric acid using a 3D graphene foam. J. Solid State Electrochem. 2018, 22 (11), 3527–3533. 10.1007/s10008-018-4047-3.

[ref91] Hassanvand Z.; Jalali F. Simultaneous determination of l-DOPA, l-tyrosine and uric acid by cysteic acid - modified glassy carbon electrode. Materials Science and Engineering: C 2019, 98, 496–502. 10.1016/j.msec.2018.12.131. 30813051

[ref92] Rezaei B.; Shams-Ghahfarokhi L.; Havakeshian E.; Ensafi A. A. An electrochemical biosensor based on nanoporous stainless steel modified by gold and palladium nanoparticles for simultaneous determination of levodopa and uric acid. Talanta 2016, 158, 42–50. 10.1016/j.talanta.2016.04.061. 27343576

[ref93] Hosseini M. G.; Faraji M.; Momeni M. M.; Ershad S. An innovative electrochemical approach for voltammetric determination of levodopa using gold nanoparticles doped on titanium dioxide nanotubes. Microchimica Acta 2011, 172 (1), 103–108. 10.1007/s00604-010-0471-5.

[ref94] Melo H. C. d.; Seleghim A. P. D.; Polito W. L.; Fatibello-FilhoIo O.; Vieira l. C. Simultaneous differential pulse voltammetric determination of L-dopa and carbidopa in pharmaceuticals using a carbon paste electrode modified with lead dioxide immobilized in a polyester resin. J. Braz. Chem. Soc. 2007, 18 (4), 797–803. 10.1590/S0103-50532007000400019.

[ref95] Shahrokhian S.; Asadian E. Electrochemical determination of l-dopa in the presence of ascorbic acid on the surface of the glassy carbon electrode modified by a bilayer of multi-walled carbon nanotube and poly-pyrrole doped with tiron. J. Electroanal. Chem. 2009, 636 (1), 40–46. 10.1016/j.jelechem.2009.09.010.

[ref96] Reddaiah K.; Madhusudana Reddy T.; Raghu P. Electrochemical investigation of L-dopa and simultaneous resolution in the presence of uric acid and ascorbic acid at a poly (methyl orange) film coated electrode: A voltammetric study. J. Electroanal. Chem. 2012, 682, 164–171. 10.1016/j.jelechem.2012.07.027.

[ref97] Arvand M.; Ghodsi N. Electrospun TiO2 nanofiber/graphite oxide modified electrode for electrochemical detection of l-DOPA in human cerebrospinal fluid. Sens. Actuators, B 2014, 204, 393–401. 10.1016/j.snb.2014.07.110.

[ref98] Kalachar H. C. B.; Basavanna S.; Viswanatha R.; Naik Y. A.; Raj D. A.; Sudha P. N. Electrochemical Determination of L-Dopa in Mucuna pruriens Seeds, Leaves and Commercial Siddha Product Using Gold Modified Pencil Graphite Electrode. Electroanalysis 2011, 23 (5), 1107–1115. 10.1002/elan.201000558.

[ref99] Reddaiah K.; Reddy T. M.; Reddy M. M.; Raghu P. Poly(Xylene Cyanol FF) Chemical Sensor for the Boost Up of Electro-Catalytic Oxidation of L-Dopa in the Presence of Ascorbic Acid and Uric Acid: A Voltammetric Study. Sensor Letters 2013, 11 (12), 2272–2281. 10.1166/sl.2013.3084.

[ref100] Mazloum-Ardakani M.; Khoshroo A.; Hosseinzadeh L. Application of graphene to modified ionic liquid graphite composite and its enhanced electrochemical catalysis properties for levodopa oxidation. Sens. Actuators, B 2014, 204, 282–288. 10.1016/j.snb.2014.07.069.

[ref101] Dhanalakshmi N.; Priya T.; Karthikeyan V.; Thinakaran N. 3D cloves bud like Gd doped ZnO strewn rGO hybrid for highly selective determination of l-dopa in the presence of carbidopa and ascorbic acid. J. Pharm. Biomed. Anal. 2019, 174, 182–190. 10.1016/j.jpba.2019.05.047. 31174129

[ref102] Anaraki Firooz A.; Hosseini Nia B.; Beheshtian J.; Ghalkhani M. Voltammetric Sensor Based on Fe-doped ZnO and TiO2 Nanostructures-modified Carbon-paste Electrode for Determination of Levodopa. J. Electron. Mater. 2017, 46 (10), 5657–5663. 10.1007/s11664-017-5625-3.

[ref103] Ghalkhani M.; Hosseini nia B.; Beheshtian J.; Anaraki Firooz A. Synthesis of undoped and Fe nanoparticles doped SnO2 nanostructure: study of structural, optical and electrocatalytic properties. Journal of Materials Science: Materials in Electronics 2017, 28 (11), 7568–7574. 10.1007/s10854-017-6448-y.

[ref104] Babu R. S.; Prabhu P.; Narayanan S. S. Highly Selective Determination of L-Dopa Using Ionic Liquid Medium Synthesized Copper Nanoparticles Modified Electrode. J. Nanosci. Nanotechnol. 2016, 16 (8), 8711–8718. 10.1166/jnn.2016.11709.

[ref105] Carvalho J. H. S.; Gogola J. L.; Bergamini M. F.; Marcolino-Junior L. H.; Janegitz B. C. Disposable and low-cost lab-made screen-printed electrodes for voltammetric determination of L-dopa. Sensors and Actuators Reports 2021, 3, 100056 10.1016/j.snr.2021.100056.

[ref106] Leite F. R. F.; Maroneze C. M.; de Oliveira A. B.; Santos W. T. P. d.; Damos F. S.; Luz R. d. C. S. Development of a sensor for L-Dopa based on Co(DMG)2ClPy/multi-walled carbon nanotubes composite immobilized on basal plane pyrolytic graphite electrode. Bioelectrochemistry 2012, 86, 22–29. 10.1016/j.bioelechem.2012.01.001. 22284852

[ref107] Daneshgar P.; Norouzi P.; Ganjali M. R.; Ordikhani-Seyedlar A.; Eshraghi H. A dysprosium nanowire modified carbon paste electrode for determination of levodopa using fast Fourier transformation square-wave voltammetry method. Colloids Surf., B 2009, 68 (1), 27–32. 10.1016/j.colsurfb.2008.09.019. 19013061

